# Staying put amidst the changing climate: lessons from older Nepalis

**DOI:** 10.1093/geroni/igaf128

**Published:** 2025-11-12

**Authors:** Liat Ayalon, Senjooti Roy, Sanju Thapa Magar

**Affiliations:** Louis and Gabi Weisfeld School of Social Work, Bar Ilan University, Ramat Gan, Israel; Louis and Gabi Weisfeld School of Social Work, Bar Ilan University, Ramat Gan, Israel; Ageing Nepal, Kathmandu, Nepal

**Keywords:** Climate change, Push–pull factors, Nepal, Non-migration, Vulnerability

## Abstract

**Background and Objectives:**

Climate change has a substantial impact on the environment, biodiversity, and human health and well-being. Individuals in the global South are particularly vulnerable to the negative effects of the changing climate, which often trigger relocation in search of better living conditions. This study relied on a phenomenological approach to understand the decision of older Nepalese to stay in place despite increasing climate threats.

**Research Design and Methods:**

Five focus group discussions with 54 Nepalese over the age of 60 (mean age 69.33 years) were analyzed using thematic analysis. All participants self-identified as non-migrants and reported experiencing severe climate change impacts.

**Results:**

Findings reveal a predominant sense of helplessness rooted in four themes: financial incapacity, perceived age-related limitations, dependency on government support, and a bleak outlook on environmental improvements. Participants expressed that poverty restricts mobility, while advanced age and place attachment reinforce their decision to remain in place. A shared belief that government intervention is inadequate further diminishes agency. Pervasive hopelessness emerged as a core barrier to migration, with many accepting their fate as unchangeable.

**Discussion and Implications:**

The study underscores how socio-economic vulnerabilities and institutional failings contribute to older persons’ inability to adapt or relocate amidst climate change. However, the findings also highlight the important role of subjective perceptions in determining one’s decision to remain in place. Addressing these barriers requires integrated policy interventions that enhance livelihood resources, strengthen institutional support, and recognize the agency of vulnerable populations to foster resilience in climate-affected communities.

Innovation and Translational Significance: This study highlights the complex interplay of socioeconomic, cultural, and institutional factors influencing older Nepalese decisions to stay in place despite the changing climate. By focusing on an often-overlooked demographic, the research highlights the important role that social institutions should play in protecting and improving the lives of older Nepalese. These findings can guide policymakers and practitioners worldwide to design inclusive, effective strategies for climate adaptation and disaster preparedness tailored to vulnerable older persons in similar contexts. While addressing objective barriers, it is essential to acknowledge the role of subjective perceptions about a lack of agency and hope.

In the past few decades, climate change has had a growing presence in our lives. According to the Intergovernmental Panel on Climate Change, climate change is characterized by more severe and frequent weather events, including extreme cold and hot weather, tornadoes, hurricanes, floods, and droughts (Intergovernmental Panel on Climate Change, 2022). Climate change has a major impact on all aspects of life, ranging from access to food, water, air, and shelter, to tremendous impacts on the environment, biodiversity, human health, and societal wellbeing (Ayalon et al., 2021; Charlson et al., 2021; Filiberto et al., 2009; United Nations Human Rights Council, 2021).

Climate change is also a major precipitator of global mobility (Almulhim et al., 2024). Although much public and academic discourse has acknowledged migration flow from the global South to the global North in search of better living conditions, within-border migration, referred to as internal migration or internal displacement, is equally or more common. Whereas migration represents a voluntary relocation (Erdal & Oeppen, 2018), displacement is considered involuntary and represents a forced act of fleeing one’s place of residence (United Nations Economic & Social Council, 1992). It has been argued, however, that the distinction between the two terms based on the voluntarism of the movement is somewhat artificial, as agency concerning relocation often occurs across a continuum. Moreover, a sense of displacement can occur even without movement (Askland et al., 2022). Thus, we rely on research that has used these varied terms, given the limited clarity in definition and usage (Erdal & Oeppen, 2018). Moreover, considering that much of the literature has not specifically focused on climate change as a main precipitator for relocation, we also rely on broader literature concerning (forced) movement from one’s home location (Abel et al., 2019).

As discussed above, climate change is a leading driver of displacement. For instance, a recent analysis based on data from Somalia between 2016 and 2018 has shown that an increase in temperature anomalies from 1 to 2°C results in a threefold increase in displaced individuals, whereas a decrease in precipitation from 50 mm to 10 mm contributes to a fourfold increase in displaced individuals (Thalheimer et al., 2023). Indeed, according to the Global Report on Internal Displacement, during 2023, as many as 75.9 million individuals were internally displaced, of whom as many as 26.4 million internal displacements occurred due to weather-related or geophysical events (Internal Displacement Monitoring Center, 2025).

Climate change is associated with various demographic changes, including rapid population aging in the most severely affected areas, given the tendency of families to leave behind older generations in deteriorating environments, while younger generations migrate in search of a better future (Hauer et al., 2024). Likewise, climate change is thought to increase inequalities both within and between countries, making rich countries/regions richer and poor localities poorer (Burzyński et al., 2021). This is attributed to the fact that migration from the global South to the global North is characterized as “brain drain,” resulting in the relocation of the most educated individuals, whereas a similar pattern is less likely to occur in the case of within-border migration. Therefore, livelihood and income loss associated with climate change is likely to be most substantial for the poorest people who live in the poorest areas, with limited ability to relocate (Burzyński et al., 2021). In addition to exacerbating inequalities, in countries where individuals have fewer opportunities to migrate, temperature increase is associated with a higher likelihood of conflicts (Bosetti et al., 2020), instigating further instability in society.

## The theoretical framework of the present study

The push–pull theory of migration explains migration at the micro-level resulting from factors that push one away from their home environment, as well as factors that pull one to a host environment. Well-known push factors include poverty, conflict, or political instability, whereas pull factors represent the reasons behind resettling in a new location with hopes for prosperity and safety (Lee, 1966). More recently, the theory was elaborated to incorporate climate and environmental issues as part of the push–pull migration considerations (Hunter & Simon, 2023). Accordingly, both sudden-onset climate events and slow-onset climate events can serve as push factors driving people away from their home location, with those living in rural areas, with livelihoods dependent on land and other natural resources, facing exacerbated vulnerabilities (Hunter & Simon, 2023).

There is some disagreement concerning the health status, resources, and resilience of individuals who relocate from their home environment versus those who stay behind. Part of this can be attributed to the use of varied terminology and, therefore, different selection criteria employed to identify the reasons and the resources behind the move (Kone et al., 2021), as well as the broader push–pull factors responsible for the move. For instance, it has been argued that when climate conditions become particularly harsh, the push factor to leave one’s homeplace increases. This may result in the inclusion of more fragile populations in relocation plans and decisions. In slightly less severe circumstances, the process of relocation might result in only the strongest and most resilient being able to leave their home community (Hunter & Simon, 2017).

## The present study

This study takes place in Nepal, a small, developing country nestled in the Himalayas, that is largely dependent on agriculture for its livelihood. Climate change has a clear and imminent presence in the lives of the Nepali people, who face multiple hurdles such as retreating glaciers, temperature increase, unpredictable rainfall, droughts, and flooding (Karki et al., 2009). Nepal is a demographically young society, with only 10% of its population over the age of 60, but its average population growth rate of 0.92% is substantially lower than the growth rate of 3.2% in the older population (Chalise, 2023), thus making Nepal a rapidly aging society.

Although Nepal’s contribution to the current climate crisis has been minimal, the country is heavily affected by it. Alterations in weather patterns are impacting multiple dimensions of people’s lives and livelihoods. The emergence of new insects, pests, and diseases that threaten Nepal’s biodiversity and impact crop production is on the rise (Rijal et al., 2022; Thorn, 2019). These in turn are contributing to a rise in morbidity and mortality, particularly among marginalized groups such as women, children, older persons and those with low socioeconomic status (Tome et al., 2022).

Seasonal climate migration is common in Nepal and has been attributed primarily to high levels of poverty and food insecurity, rather than to climate change (Gautam, 2017). Likewise, high-caste Hindus are less likely to migrate than people of lower castes, although environmental changes have also been associated with out-migration (Massey et al., 2010). While Nepal is highly dependent on remittances by labor migrants (Dhakal & Paudel, 2023), non-migration, despite environmental challenges, has been attributed to strong place attachment; existing, accumulated social capital, which helps in managing challenges; and confidence in local adaptation and survival strategies (Bhusal et al., 2021). Although Nepal has several climate policies in place, they have been criticized for their limited operational clarity, poor inter-ministerial coordination, absence of clear implementation mechanisms, limited stakeholder capacity, inadequate decentralization, and insufficient access to finance and technology (Maharjan & Maharjan, 2017).

In our inquiry, we specifically aimed to learn about the experiences of older Nepalese. Older persons represent the population that is most susceptible to the effects of climate change (Adams et al., 2011, 2021). Due to their weakened immune systems, pre-existing medical conditions, and impaired physical and cognitive functioning, many older persons are vulnerable to the negative effects of climate change (Prina et al., 2024). Older persons are also more likely to depend on assistance for daily care, a crucial aspect of evacuation planning during natural disasters that additionally places older persons at a greater risk for impaired health and increased mortality following climate exposure (McGuire et al., 2007). Moreover, as already noted, it is usually younger persons who leave deteriorating environments in search of better futures, while older persons stay behind, facing climate-related challenges on their own (Hauer et al., 2024). The present study is focused on older persons who self-identified as being highly impacted by the changing climate. In their reports, they addressed the degradation of the land and its impact on various aspects of their lives, including livelihood, food, and health insecurity. Nonetheless, they elected to remain in their place of origin, rather than migrating elsewhere in search of better living conditions. This study aims to understand the reasons for this, relying on a phenomenological perspective, which aims to present the voices of older persons as authentically as possible.

## Method

### Participants in the study

Funding for this research was provided by a grant from the Israel Science Foundation (ISF 217/20). Ethical approval was obtained from the Ethics Committee of the School of Social Work at Bar Ilan University, Israel (approval number 150924288, dated 15.9.24). A total of 54 individuals participated in focus group discussions to share their experiences of the changing climate. Eligibility criteria included being a non-migrant over the age of 60. As the study aimed to explore the lived experiences of older persons within the context of climate change, we selected an area that is vulnerable to slow as well as acute climate events. Most of the population in the selected area belonged to indigenous, highly marginalized, and minority communities. Approximately 43% of residents live below the poverty line, with an annual income of less than USD 150; agriculture is their primary source of livelihood (Rakshirang Municipality, 2025). All participants self-identified as non-migrants, although some had moved short distances within their municipality. The participants’ average age was 69.33 years (ranging from 60 to 89 years). Of these, 23 (42.59%) were women, and 51 (around 94%) had no formal education. Detailed demographic information can be found in Table 1.

**Table 1.** Participants’ characteristics (*N *= 54).

Characteristics	*N* (%)
**Gender**	
** Male**	31 (57.40)
** Female**	23 (42.59)
**Age group**	
** 60–70 years**	30 (55.55)
** 71–80 years**	21 (38.88)
** 81–90 years**	3 (5.55)
**Education**	
** No formal education**	51 (94.44)
** Less than high school**	3 (5.55)
**Marital status**	
** Single**	5 (9.25)
** Married**	26 (48.14)
** Divorced**	1 (1.85)
** Widowed**	22 (40.74)
**Religion**	
** Hindu**	16 (29.62)
** Christian**	15 (27.77)
** Buddhist**	23 (42.59)

### Procedure

The study was undertaken in Rakshirang Rural Municipality, which comprises around 5,044 households. This is a rural administrative region located in the western and northern parts of the Makwanpur district within Bagmati Province, approximately 150 km from Kathmandu, Nepal’s capital. It is the most remote municipality in Makwanpur and was selected due to its vulnerability to long-term climatic shifts such as droughts, temperature rise, and rainfall variability, which have impacted agricultural productivity and water availability. Additionally, the area is susceptible to flash floods, landslides, storms, cloud bursts, and lightning strikes, especially during the monsoon season. Participants were recruited with the support of the mayor of the municipality, village leaders, and local self-help groups. Two Nepalese field researchers who were familiar with the geographical layout of the area and had contacts with community leaders and well-connected community members recruited participants via informal networks and snowball sampling. Information about the study was communicated by word-of-mouth to community members and leaders who helped identify suitable participants for the study. The field researchers conducted five focus groups, each comprising 9 to 11 participants. The sample size was determined in line with the quantitative part of the study, which is not reported here. The relatively large sample size also allowed us to ensure adequate representation of both men and women. The decision to form focus groups rather than individual interviews stemmed from their financial feasibility but also from the fact that such a methodology allows for approaching the group as a whole and obtaining a consensus about its experiences (Acocella, 2012). Key topics covered in these discussions included environmental degradation (e.g., “think back to the time before you started experiencing the impacts of climate change and environmental degradation in your daily lives”), migration opportunities (e.g., “share why you have decided to continue to live in your home and land instead of migrating to other places for better prospects”), food insecurity (e.g., “describe how climate change and environmental degradation has impacted your dietary habits, choices, and preferences”), social networks (e.g., “who supports you the most? How do you spend your leisure time?“), and health and healthcare services (e.g., “share how climate change and environmental degradation in your area has impacted your physical health.”). The sessions commenced with general questions, such as, “Why have you decided to continue to live in your home and land instead of migrating to other places for better prospects?” and gradually became more specific, such as, “How safe do you feel living in this area as your environment changes around you?” For more information about the interview guide, refer to supplementary material. All focus group discussions were held in locations of convenience for the participants and were conducted in the local language, *Nepali*. All sessions were audio recorded, transcribed, checked for accuracy, and translated into English.

### Analysis

We performed a thematic analysis following Clarke and Braun’s (2017) approach. The first author read and re-read each interview to become thoroughly familiar with the data. Initially, descriptive codes were assigned, which were followed by more interpretive coding. The second author also reviewed the interviews, familiarized herself with the data, and conducted preliminary coding. This analysis was conducted using separate Word documents, in which codes with corresponding extracts from the focus groups were organized. The first two authors subsequently shared and combined their codebooks to reach a consensus concerning the main themes that emerged from the focus group discussions. Following this, the themes were systematically organized to elicit a comprehensive understanding of the participants’ migration-related experiences and decision-making. While various themes were identified—such as food insecurity, care concerns, and healthcare access—this study focuses on reasons for non-migration from the perspective of older Nepalese. To capture subtle nuances, we alternated between descriptive and interpretative coding. Selected direct quotes from the data are provided to illustrate the main themes identified.

### Trustworthiness

All three authors reviewed both the data and its analysis. The first two authors independently engaged in data analysis and discussed their decisions concerning thematic classifications. The third author, a Nepalese resource person and collaborator on this project, provided her perspective on the study findings. She also engaged in the development of the interview guide and in methodological decisions regarding the suitability and efficacy of focus groups for data collection. Additionally, we relied on reflexivity, acknowledging the fact that two of the authors who led the project were non-Nepalese, whereas data collection had been undertaken by Nepalese field researchers. To overcome some of the challenges associated with implementing a Western methodology in a non-Western culture, we obtained continuous feedback from our Nepalese collaborator (third author) during all stages of the study, including its very preliminary inception and conceptualization.

## Findings

The overarching theme that captures the phenomenon of non-migration among the participants, despite experiencing both slow and sudden climate events, is that of a lack of agency and a sense of helplessness. Within this broad theme, four sub-themes address a variety of reasons for non-migration. First, participants stressed their limited financial situation as a detriment to their ability to migrate to better environments. Second, participants felt limited by their age, as they believed that there was no point in migrating elsewhere and leaving their home environment at an advanced age. Third, participants believed that the government should assume responsibility for their affairs, thus representing a complete dependence on external sources for assistance. And fourth, a general worldview held by the participants was that conditions would be no better elsewhere, regardless of one’s migration decisions and actions, thus representing complete surrender to the deteriorating circumstances of their lives and their self-perceived inability to imagine a better future.

### Subtheme 1: Too poor to move

The participants in this study represent individuals of low socioeconomic status. They reside in rural areas and work in agriculture. As such, they represent a group that is highly dependent on land and natural resources and therefore greatly impacted by the changing climate. The following are statements by participants who expressed a wish to migrate, while acknowledging that, due to limited finances, relocation was impossible.

…we want to move to a safer, better location too, but we don’t have many options, I don’t have the capital [money] to move. Lack of finance compels us to live here.

Without resources, wanting or needing to migrate is pointless. We don’t have the privilege to move. Neither us nor the government has been able to help us.

A different participant stressed the fact that it was not attachment to the land that confined him to his current location, but rather the understanding that he could not afford to relocate, given his limited means:

I’ve been living here since 1957. But that’s not the reason for not leaving this place. We haven’t migrated because we are unable to: 0.03 acres [of] land now cost 12 to 15 lakh Nepali Rupees [approx. USD 8,700-11,000] in this area, let’s not even talk about Kathmandu. Let’s not even discuss Narayanghat, Birgunj, Manahari [other cities]. What can we do? The prices are very high.

This participant’s views were corroborated by a fellow participant who had also contemplated migration but changed his mind in light of financial constraints:

If we sell our land and migrate to another place, then it won’t be enough to buy even the land for a toilet. That’s why we are here.

One participant summarized the situation succinctly, seeing no prospect of migration as a strategy to improve her situation:

We are surviving anyhow we can. What can we do if we’re in a place like this? We don’t have money to migrate to a better place. What can we do, we the poor only have to stay here.

This sub-theme illustrates how even strong “push factors” driving migration may be insufficient to overcome financial barriers, especially for those with meagre resources and limited prospects for improved livelihoods. As such, migration was viewed as a privilege borne out of necessity but only undertaken by those with sufficient means.

### Subtheme 2: Too old to move

A major reason for not considering a better future elsewhere lay in the participants’ perception of themselves as older persons who were incapable of relocating due to their advanced ages. In the following statement, one participant’s perceived inability to relocate due to her age rendered her life, safety, and livelihood completely dependent on younger people:

We’re old and we have no options. We can’t run so we have to stay thinking, “It’s okay if I die.” I feel like if someone young saves me [during a natural disaster] I can live. Otherwise, I don’t think I’ll be able to make it out.

A different participant reported a similar experience, stating she had no option to migrate simply because of her advanced age:

We have to live here anyhow. There is no option for us to migrate at this age. So, we’re preparing to live here in any situation whether sad or happy.

Another participant also acknowledged his advanced age and linked it to his inability to migrate. He additionally mentioned place attachment as a reason to stay in place, despite the multiple challenges brought about by climate change: “It’s too late for me to move now, if we move now, we’ll miss this place.”

The following response illustrates once again the strong tie between one’s perceived advanced age, limited financial situation, and inability to relocate due to lack of viable alternatives:

This is the place where I have to spend the rest of my life even with all the rain and flood. In this condition where can we old people go? If an earthquake occurs, do you think we can get out of the house in time? The same happened back in 2004 and 1994. All places are the same for the poor. Wherever we go, we can’t eat without our own hard work. It’s the same if we go to the city. It’s the same if we go to Kathmandu. Somebody must be feeling like going there. But now they say even Kathmandu City has drowned. What to do?

As age, financial limitations, place attachment, and lack of prospects converged and collided to create diverse circumstances, participants surmised that adapting-in-place to the best of their abilities was perhaps better than moving to unfamiliar environments and starting anew at advanced ages.

### Subtheme 3: Our future is in the hands of the government

Several participants expressed the belief that the government should take care of their welfare as they were older adults residing in ecologically vulnerable locations. This belief reflected passive acceptance of current circumstances, with no clear vision for a future, especially given the government’s lack of initiative for change:

…climate change has made it risky to live here, even the dam built on elevated grounds has proven to be unsuccessful…this is a huge issue for the safety and security of the village. We’re hopeful that the State will do something for us, although we know we won’t get help, but still, we’re hopeful at heart. This village is unsafe due to the geographical shape of the land too; we hope to move to another place if the government can manage it. The living conditions, working conditions, weather, etc. are all good here. In monsoon, however, it is tough to live here, the river and rivulets’ water level is so unpredictable that they can wash away our houses and properties. This year the rain impacted east and west. I guess it is our turn next.

A different person argued for the inability, or perhaps the unwillingness, of the State to help, thus not only reflecting a lack of personal self-efficacy but also a broader perception of the system as failing to assist:

Even the State can’t help us. My house got destroyed in 2015’s earthquake. This is my nephew’s house, Sir. My house is a little far, but it is very similar to this one. I couldn’t reconstruct it, so my sister reconstructed. I have the same house with one room each for my sister and me. We’re surviving in it. We are poor, what else can I say?

The lack of faith in the State was shaped by the participants’ past experiences as even 10 years post a natural disaster in 2015, the State is yet to compensate them for their losses.

Sir, after the earthquake of 2015, we were promised compensation of fifteen hundred thousand [Nepali Rupees] for each house, but the State fooled us with three hundred thousand. We’re realizing [recovering] it gradually. Now, we still have to receive about three hundred and fifty thousand.

The following statement showcases the compulsions born out of poverty, which place participants at a disadvantage as their need for help could potentially clash with the consequences of receiving help. This participant expressed fear of forced migration and a belief that government assistance would not be sufficient to improve their lives:

We’re also worried that the government might move us from here to someplace similar or worse than this. I think the poor will always be left [behind]. Just managing the waterflow of the river Manohari could be a huge relief. It can be done either by nets or something [else]. The roads are all dug up and with the rain all that washes over. We’re fearful of flooding every time it rains.

These sentiments reflect a common occurrence in developing countries where residents of a locality may be acutely aware of both the problem and the potential solution and yet lack the ability to implement changes owing to government apathy. Often, such a wait may span years as governments invest funds in more urban locales while ignoring rural areas.

### Subtheme 4: Things will not get better

Whereas the first three sub-themes describe reasons for perceived helplessness and lack of agency, the fourth sub-theme simply expresses lack of hope for a change. According to the following statements, there is no other option, because all options will pose similar threats and challenges:

I don’t know where to go. Kathmandu is a better place than this, but it too is struggling with floods.

Regardless of damage caused by an earthquake [and] flood, we are compelled to tolerate everything and live in our birthplace. We were born here, and we will die here. Even the place we want to move [to] is hot. If we move toward the hill, the cold becomes unbearable. If we move down to the Tarai [plains], the heat becomes unbearable. So, being in the middle is perfect for us.

The participants’ lack of hope also stemmed from their past experiences as some participants had migrated within their municipality after enduring natural disasters, only to find that conditions were not very different. Their beliefs were further reinforced by the news that they heard on the radio and other media outlets about climate change impacting all places adversely.

We were chased by floods and landslides from one place, and now we’ve come here. We can’t even trust this place. Now that we are here, we are just living off whatever happiness we can find. Anyway, this place is also unpredictable and I have fear about it.

…with time a lot has changed around here. I’ve heard from scientists over the radio that the heat percentage is getting up, that is what I feel like too, Sir.

The participants’ sense of hopelessness was also influenced by thoughts about death and the futility of chasing better options at a stage when “*it is already time for us to leave for another world*”:

It’s time to leave this earth, why would we move [to] some other place? We’re preparing to live here.

Where to go now? No, we will not go anywhere. I will stay here forever…there is peace after death.

This theme highlights how migration decisions are shaped by various physical, emotional, and social circumstances of individuals. It shows how desire for better lives can be dwarfed by the magnitude and universality of the climate crisis, rendering people helpless and hopeless.

## Discussion

Nepal is a developing country, primarily reliant on its agricultural economy and highly impacted by climate change (Karki et al., 2009). This study focuses on rural Nepalese older persons who, in many ways, are amongst the most vulnerable individuals worldwide, given their high dependence on the land in a poor country with limited resources. The findings highlight the dearth of opportunities available to older Nepalese who confront the changing climate with relatively constrained internal and external resources.

The push–pull theoretical model, which was recently elaborated to explain migration in the context of climate change (Hunter & Simon, 2023), was highly relevant in explaining respondents’ decisions to stay in place. The participants in the present study described multiple push factors associated with the changing climate and growing life and livelihood insecurity. However, they did not identify pull factors to encourage their relocation. Hence, the findings suggest that a clear push–pull balance must occur for actual migration to take place.

A general disbelief in a better future was expressed by the respondents, illustrating a clear acknowledgment of the challenges associated with relocation and the limited resources they have. “Learned helplessness” is a term used to describe a sense of powerlessness and inability to effect desired changes resulting from repeated exposure to uncontrollable stressful situations (Seligman, 1972). In the case of older Nepalese, there are many reasons why learned helplessness has developed. These individuals are constantly faced with sudden and slow climate events over which they have no control. They have limited personal resources in the face of climate challenges that threaten their livelihoods and safety and are therefore unable to effect changes even if they desire to do so. Additionally, based on their reports, the institutions responsible for their protection have also failed them, leaving them with minimal opportunities to handle the severe climate and environmental changes that have occurred over the years.

An important observation pertains to the participants’ beliefs that there is little to gain by striving for better circumstances in the material world when true peace can only be experienced after death. As most participants were either Hindu or Buddhist, this belief is likely tied to the concepts of *karma* (cause-and-effect) and rebirth/reincarnation. In Hinduism, this refers to the belief that the soul reincarnates in successive lifetimes to fulfill specific purposes. Accordingly, all individuals experience the “effects” (*karma-phala* or the “fruits of *karma”*) of previously performed actions (*karma*). Buddhism also teaches *karma* and rebirth but understands it as the continuation of mental and karmic processes without an eternal soul. Both spiritual traditions view life as marked by suffering, with desire as its root, and see lasting peace as attainable only through liberation from the cycle of rebirth (Lin & Yen, 2015; Medhananda, 2025). Therefore, the participants’ resignation to the suffering of hardships, combined with a lack of desire to improve their circumstances, while waiting for peace on the other side of this material world, most likely stems from these religious beliefs. Instead of looking for a temporary way out in this lifetime, especially at advanced ages, they aspire for permanent peace after death. Perhaps this belief also allows them to justify their lack of agency or learned helplessness with respect to improving their circumstances.

A major reason for the fact that the older participants of this study had remained in their place of origin rather than relocating was their limited financial resources. Many of them did not see a point in migrating, as their insufficient means would not allow them to improve their situation elsewhere. Given their financial constraints, respondents expressed the expectation that the government would take care of their affairs. In many ways, the findings illustrate how government inaction can turn present natural hazards into future disasters. Whereas hazards are certainly natural, reflecting substantial changes in the climate and the environment, they turn into unnatural disasters due to the failure of institutions to protect the most vulnerable (Puttick et al., 2018). This failure was clearly described, as well as feared, in many of the participants’ experiences.

Another reason for the lack of mobility was the participants’ self-perceptions as being too old. Although the age of 60 is defined as “old” by the United Nations (United Nations High Commissioner for Refugees, 2025), old age may start earlier, given the greater physiological susceptibility of individuals in the developing world (Ashirifi et al., 2023). This study illustrates how one’s subjective perceptions of old age can shape one’s perceived ability to relocate in the face of challenges brought by climate change, thus providing further evidence of the relationship between subjective age and sense of self-efficacy (Stephan et al., 2011).

Interestingly, in addition to attributing their inabilities to their age, respondents also stressed the role of attachment/lack of attachment to place, which in their view seemed connected to one’s age. Indeed, research has shown that attachment to place is particularly pronounced in the case of older persons (Lebrusán & Gómez, 2022), suggesting that this might be a reason for lack of mobility among this population (Rabbani et al., 2022). This has been true even for Nepal, with research showing that place attachment is associated with non-migration. However, others have argued that place attachment does not always explain future non-migration decisions (Mallick, 2023). This study also did not find place attachment to be a substantial factor in keeping people in place, though it was mentioned several times by respondents, especially in conjunction with their advanced age. This could be attributed to the high levels of poverty and substantial exposure to environmental challenges experienced by the present sample. Moreover, although not reported in this study, when analyzing the data, we also found the disruption of social support and community relations, which may further hamper people’s sense of place attachment (Ayalon & Roy, manuscript submitted for publication).

Despite its strengths, the study has several limitations. First, the study was conducted in a relatively small geographic area (one municipality) in Nepal and must be interpreted within that context. The Himalayan range is inhabited by people living at various altitudes, leading to distinct lived experiences. Additionally, cultural and linguistic barriers may affect the interpretation of the findings, as the data collection was conducted by local Nepalese field workers, but data analysis and write-up were conducted by people who are foreigners to the culture, though input from local team members was provided. In addition, the present sample was relatively poor, coming from a rural area that largely depends on agriculture for income and sustenance. Future research will benefit from learning about the experiences of Nepalese of various socioeconomic positions to better understand intersectionality. Likewise, further analysis by gender is justified, given research that has shown gender differences in the impact of climate change (Li et al., 2025). It is also beneficial to examine the impact of climate change on younger non-migrants to understand whether the constraints that keep older persons in place are different from those that keep younger persons in place.

### Implications for policy and practice

Despite its limitations, the study has important implications for policy and practice. First, it is a clear testimony to the important role that institutions must play in protecting the lives and livelihoods of the most susceptible segments of the population, such as older persons in rural Nepal. In the present study, these institutions have failed older persons and have left them to take care of themselves. However, the study also shows that the personal resources possessed by older Nepalese are quite limited, resulting in a heightened sense of helplessness. Although learned helplessness is certainly not an adaptive strategy, there are also dangers to enhancing one’s sense of self-efficacy in the face of objective limitations (Peleg & Ayalon, 2015). Therefore, it is important to address not only subjective perceptions but also objective limitations, which impair the ability of older Nepalese to change their lives for the better. Empowering individuals is desirable if indeed they have the power to change their lives. However, in this study, it was clear that older Nepalese need more than just empowerment. They need financial and structural support to help them deal with the changing climate and environment. It is also important to note that the participants’ belief in their inability to make a difference has developed over time, shaped by repeated experiences of environmental disasters and neglect by authorities. Hence, it is important that assistance is provided throughout one’s lifespan, as and when people experience climate events, rather than only in old age. Over time, this will allow them to develop greater resilience and enable long-term planning. Consequently, they may feel more empowered to handle challenging situations as they grow older. The people interviewed in this study largely rely on subsistence agriculture for sustenance and livelihood. This is a much-needed source of livelihood for Nepal. Hence, it is important to further support them financially as well as via programs that will help them adapt to and mitigate the challenges associated with the changing climate more effectively. This follows the logic of providing people with a fishing rod, rather than a fish.

## Supplementary Material

igaf128_Supplementary_Data

## Data Availability

Data are available upon request, subject to ethical approval. The study was not pre-registered.
