# Supplementary material for: Staying put amidst the changing climate: lessons from older Nepalis
Source: Innov Aging. 2025 Nov 12;9(12):igaf128. doi: 10.1093/geroni/igaf128 (PMC12705134; doi:10.1093/geroni/igaf128)
Supplement: igaf128_Supplementary_Data [file igaf128_supplementary_data.zip › innage suppl Ayalon, Roy, & Magar.docx]

***Innovation in Aging* Supplementary Material: Ayalon, Roy, & Magar. Staying put amidst the changing climate: Lessons from older Nepalis.**

**Focus Group Interview Guide**

***(Probes are in italics)***

- Welcome and introduction of researcher
- Introduction to study/topics to be discussed
- Guidelines/ground rules for FGD
- Brief introduction of participants

**Topic 1: Environmental changes**

1. Please share why you have decided to continue to live in your home and land instead of migrating to other places for better prospects.
2. Please think back to the time before you started experiencing the impacts of climate change and environmental degradation in your daily lives. What are some predominant memories and emotions that you associate with that time in relation to your environment?
3. What are some predominant emotions that you currently experience in relation to your environment?
4. What are some factors that would make you consider migrating to another place?
5. How safe do you feel living in this area as your environment changes around you?
6. How do you feel about ageing and possibly spending the last part of your life amidst climate change impacts in your area, especially in relation to those who have migrated elsewhere for better lives?

**Topic 2: Social support**

1. Once again, please think back to the time before you started experiencing the impacts of climate change and environmental degradation in your daily lives. Please describe your social support network from that time (*size, members, physical proximity, availability, dependability, frequency of contact, who supports you the most?*). How do you spend your leisure time?
2. Now please think about your current circumstances and describe your social support network. Who supports you the most? How do you spend your leisure time?
3. Please describe your current living arrangements (*type of residence, household size, composition, proximity to family and friends*)? What are some pros and cons of this arrangement?
4. Please describe what caregiving arrangements and support systems are in place for you as you continue to age. Conversely, if you are the one providing care, please share experiences/challenges/difficulties that you face.

**Topic 3: Food Security**

1. Please describe how climate change and environmental degradation in your area has impacted your ability to cultivate (*grow crops/raise livestock/farm fish)* and buy food.
2. Please describe how climate change and environmental degradation has impacted your dietary habits, choices, and preferences. What are some adjustments that you have had to make to your diet (*due to unavailability of certain food items/cost of food/difficulty in procuring fuel/unavailability of clean water)*?
3. Please discuss any age-related dietary needs that are currently not being met. How do you handle this?
4. How do you feel about the future stability of food availability in your current place of residence.

**Topic 4: Health and Wellbeing**

1. Please share how climate change and environmental degradation in your area has impacted your physical health.
2. Please share how climate change and environmental degradation in your area has impacted your mental and emotional health (*how do you feel most of the time in relation to your environment*?)
3. Please share any concerns that you may have about the healthcare facilities that you currently access (*distance from the nearest healthcare facility; the availability of doctors, nurses, medicines, facilities; frequency of visit*).
4. As an older person, what are some health-related needs that you have that are not being currently met?

**Closing:**

- A summary of the 4 discussions, if possible.
- **Question:** Is there anything else about the topics that we have discussed today that you think we should know but have not asked?
- Conclusion of session, thanking everybody for their participation + refreshments
